# Calcium-sensing receptor and calcium kidney stones

**DOI:** 10.1186/1479-5876-9-201

**Published:** 2011-11-22

**Authors:** Giuseppe Vezzoli, Annalisa Terranegra, Francesco Rainone, Teresa Arcidiacono, Mario Cozzolino, Andrea Aloia, Elena Dogliotti, Daniele Cusi, Laura Soldati

**Affiliations:** 1 Nephrology and Dialysis Unit, San Raffaele Hospital, Vita Salute University, Milan, Italy; 2 Department of Medicine, Surgery and Dentistry, San Paolo Hospital, Università degli Studi di Milano, Milan, Italy

**Keywords:** calcium, kidney stones, calcium-sensing receptor

## Abstract

Calcium nephrolithiasis may be considered as a complex disease having multiple pathogenetic mechanisms and characterized by various clinical manifestations. Both genetic and environmental factors may increase susceptibility to calcium stones; therefore, it is crucial to characterize the patient phenotype to distinguish homogeneous groups of stone formers. Family and twin studies have shown that the stone transmission pattern is not mendelian, but complex and polygenic. In these studies, heritability of calcium stones was calculated around 50%

Calcium-sensing receptor (CaSR) is mostly expressed in the parathyroid glands and in renal tubules. It regulates the PTH secretion according to the serum calcium concentration. In the kidney, it modulates electrolyte and water excretion regulating the function of different tubular segments. In particular, CaSR reduces passive and active calcium reabsorption in distal tubules, increases phosphate reabsorption in proximal tubules and stimulates proton and water excretion in collecting ducts. Therefore, it is a candidate gene for calcium nephrolithiasis.

In a case-control study we found an association between the normocitraturic stone formers and two SNPs of CaSR, located near the promoters region (rs7652589 and rs1501899). This result was replicated in patients with primary hyperparathyroidism, comparing patients with or without kidney stones. Bioinformatic analysis suggested that the minor alleles at these polymorphisms were able to modify the binding sites of specific transcription factors and, consequently, CaSR expression.

Our studies suggest that CaSR is one of the candidate genes explaining individual predisposition to calcium nephrolithiasis. Stone formation may be favored by an altered CaSR expression in kidney medulla involving the normal balance among calcium, phosphate, protons and water excretion.

## Calcium nephrolithiasis: one or more disorders?

According to modern criteria, calcium nephrolithiasis can be considered as a multifactorial disorder, including different groups of patients, homogeneous for their final phenotype but heterogeneous for their intermediate phenotypes and pathogenetic mechanisms. Therefore, it appears as a complex disease that may develop changeable clinical manifestations in urine composition, bone involvement, stone composition, age at onset and other intermediate phenotypes [1,2]. Both genetic and environmental factors may increase susceptibility to calcium stones, but the weight of each factor may vary in different patient groups [1]. Therefore, it is crucial to characterize and properly define the patient phenotype in order to distinguish homogeneous groups of stone formers.

Studies on families and twins showed the genetic determination of nephrolithiasis. Stone formers were more common among the first-degree relatives of stone-forming patients than healthy individuals. Concordance for stones was greater among monozygous than dizygous twins. In both studies, hereditary of calcium stones, defined as the proportion of the phenotipic variance depending on genes, was approximately 50% [3-6].

Family analysis also showed that the transmission pattern of calcium stones is not mendelian, but complex and polygenic, although 3 decades ago the first studies proposed an autosomal dominant hereditary transmission [3,4]. The pattern of the hereditary transmission has not been ascertained yet, but it is likely that multiple genes having a small causal effect may contribute to increase susceptibility to stones. Effects of these genes could be additive and calcium kidney stones may develop when the sum of their effects exceeds the threshold of stone formation in urine [2,6]. This hypothesis does not imply the involvement of "major genes" having a necessary and predominant role in stone formation [7,8].

## Genetic approach to calcium stones and the calcium-sensing receptor

In our preliminary clinical approach to the disease, we evaluated the association of calcium nephrolithiasis with the polymorphisms of 10 candidate genes, selected on the basis of the knowledge of the possible role of their products in stone formation.

We selected genes of two calcium pumps of the plasma membrane (ATP2B2 and ATPB1), the anion channel (SLC4A1), the calcium-sensing receptor (CaSR), a phosphate carrier (SLC34A1), the vitamin D receptor (VDR), two dicarboxylic acid carriers (SLC13A1 and SLC13A2), the lysosomial H-pump (ATP6N1B), the calcium channel of the distal tubule (TRPV5). We chose 2-6 biallelic polymorphisms on each gene (Unpublished data). We only found an association between calcium stones and rs1501899 (G > A), a SNP located on the first intron of the CaSR gene. The minor allele A at rs1501899 was more frequent in stone formers than healthy subjects [9].

CaSR gene (chr. 3q13.3-21) encodes for a protein of 1078 aminoacids present in the plasma membrane as a dimer. CaSR is a member of the G-protein coupled receptors and its structure has 3 different domains [10,11]. The extracellular domain (612 aminoacids) binds extracellular calcium through its multiple negative charges; the transmembrane part (250 aminoacids) has 7 membrane-spanning domains; the intracellular tail (216 aminoacids) interacts with the G-proteins and filamin A to translate within the cells the signal produced by the extracellular calcium binding [12,13]. The CaSR gene has two different promoters (P1 and P2), whose functional differences are not yet known. Each promoter contains responsive elements to vitamin D receptor and interleukin 1 that stimulate CaSR gene transcription and CaSR expression [14,15].

The CaSR binds extracellular calcium with a low affinity (EC50 3 mM in vitro), however, the binding is a highly cooperative process (Hill coefficient is around 3) allowing the CaSR to function as a sensitive detector of extracellular calcium [16]. These properties render serum calcium (1.2 mM in healthy people) the main activator of CaSR in the human body and enable human cells to modulate their function on extracellular calcium. This process takes mainly place in parathyroid and kidney tubular cells regulating calcium concentrations in extracellular fluid. Thanks to its sensitivity to multiple cations, the CaSR behaves as a sensor of extracellular ions: employed as a salinity receptor in fishes, it evolved as a regulator of calcium homeostasis in humans [10,17,18].

CaSR stimulates distinct intracellular signaling pathways according to the different G proteins interacting with it. Its interaction with the Gq protein activates the C phospholipase, which releases inositol triphosphate (IP3) and diacylglycerole (DAG) from phospholipids. IP3, after binding to its specific membrane receptors, stimulates calcium release from the stores in the endoplasmic reticulum, while DAG stimulates the protein kinase C (PKC) activity [12,19]. CaSR interaction with protein Gqα activates phospholipase A2 and stimulates the production of arachidonic acid and 20-hydroxy-eicosa-tetra-enoic acid (HETE) [20]. Furthermore, CaSR interaction with Gi protein inactivates phospholipase A2 and inhibits cAMP production. Therefore, through these and other pathways, CaSR may influence cell function, but also cell proliferation and gene expression [21,22].

CaSR is a ubiquitous protein, mostly expressed in the parathyroid glands and in renal tubules, especially distal tubules [23]. Thanks to its presence, parathyroid cells regulate the PTH secretion according to the serum calcium concentrations. The increase in extracellular calcium concentrations stimulates CaSR and inhibits PTH secretion and cellular proliferation [10,16]. The opposite occurs when the serum calcium concentration decreases, thus explaining the proliferation of parathyroid cells after prolonged periods of hypocalcemia and secondary hyperparathyroidism, like those occurring in chronic renal failure [24].

### CaSR in the kidney

**Figure 1:**
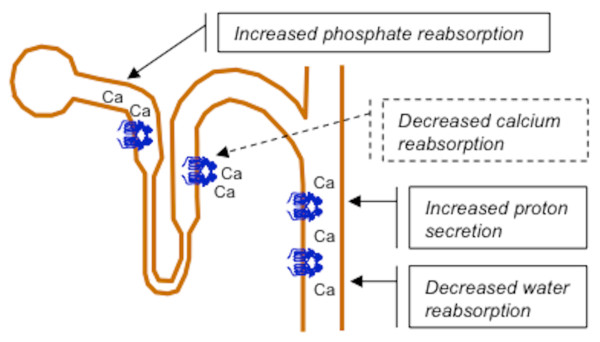
**CaSR is located on the basolateral membrane of the tubular cells in the thick ascendent limb of Henle loop and in the distal convoluted cortical tubule, where it senses serum calcium and reduces calcium reabsorption when activated by serum calcium increase**. In proximal tubules and collecting ducts it is located on the apical membrane and its activation by the increase in tubular fluid calcium may protect against calcium-phosphate precipitation.

In the kidney, the CaSR performs different tasks depending on the various tubular segments in which it is located (figure 1) [25]. It is expressed on the luminal membrane of the proximal tubular cells where it senses the increase in calcium luminal concentrations and inhibits cAMP production induced by PTH [23,26]. In proximal tubules, PTH causes phosphate excretion by internalization and degradation of phosphate reabsorption carriers (NPT2c and NPT2a) in subapical vesicles derived from brush border. In-vitro microperfusion studies of single mouse proximal tubules showed that CaSR limits PTH activity and decreases urinary loss and luminal concentrations of phosphates [27].

**Figure 2:**
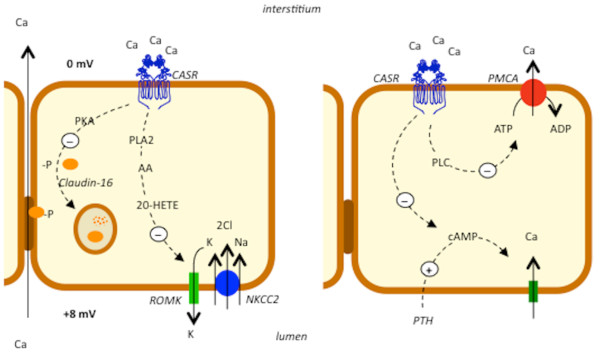
**CaSR is expressed on the basolateral membrane in the thick ascending limb of Henle loop (left panel) and in the cortical distal convoluted tubule (right panel)**. In the thick ascending limb of Henle loop, CaSR inactivates the luminal potassium recycling through the ROMK channels. This effect dissipates the positive luminal electrical potential that is the driving force for passive paracellular calcium reabsorption. In this nephron segment, CaSR also inhibits the phosphorilation of caludin-16, which can be expressed in tight junctions only after phosphorilation. The absence of claudin-16 reduces the tight junction permeability to calcium and magnesium and, as a consequence, passive calcium reabsorption. In the cortical distal convoluted tubule, CaSR reduces calcium pump activity (PMCA) and calcium active reabsorption. PLA2 is phospholipase A2, AA is arachidonic acid, 20-HETE is 20-hydroxi-eicosa-tetraenoic-acid, PLC is phospholipase C, PKA is protein kinase A. Split lines express the enhancing (plus sign) or inhibitory (minus sign) pathways activated by CaSR.

CaSR is expressed on the basolateral membrane of the thick ascending limb of Henle loop [26]. In this tubular segment, a sodium-potassium-chloride carrier (NKCC2) couples the inward transport of these three ions through the apical membrane. The activity of NKCC2 is sustained by the sodium-potassium-pump in the basolateral membrane and by the low-conductance potassium channels (ROMK) in the apical membrane [21]. ROMK allows potassium ions recycling from cytoplasm into the tubular lumen, thus sustaining the positive luminal charge of the electric gradient between interstitium and tubular lumen. This electric gradient becomes the driving force for paracellular reabsorption of sodium and calcium [21]. In in-vitro studies on microdissected or isolated microperfused tubules from laboratory animals, CaSR activation by serum-interstitial calcium enhanced the intracellular production of arachidonic acid and HETE that can inactivate the potassium ROMK channel and the Na-K-2Cl cotransporter [20]. The inactivation of these carriers inhibited the passive reabsorption of sodium, potassium and chloride and blocked the potassium recycling to the lumen through the specific ROMK channel [20,21]. This mechanism dissipates the positive luminal electrical potential generated by potassium recycling and decreases the passive calcium reabsorption (figure 2). In thick ascending limbs isolated from dogs, CaSR also inhibited the phosphorilation of claudin-16 through a mechanism mediated by the decrease in the protein kinase A activity. The unphosphorilated form of claudin-16 was not expressed in tight junctions and its absence reduced the tight junction permeability to calcium and magnesium, thus amplifying the calciuric effect of CaSR [28]. In cortical segment of the thick ascending limb, explored in mice by single tubule microperfusion experiments, CaSR decreased the PTH-dependent passive calcium reabsorption through the apical membrane by antagonizing the PTH-stimulated cAMP production (figure 2) [29].

In the distal convoluted tubule, CaSR is located on the basolateral membrane of tubular cells and in cultured dog cells from this tubular segment CaSR was found to reduce the active calcium reabsorption by interfering with the calcium pump function through a phospholipase C dependent mechanism. The signaling pathway for this effect requires activation of G_q _proteins [30].

In the collecting duct, CaSR is expressed on the apical membrane of the principal and intercalated cells [23,31]. In cultured mouse principal cells, CaSR altered the trafficking of aquaporin 2 (AQP2) and reduced the urine concentrating ability by antagonizing vasopressin activity and the cAMP-dependent activation of protein kinase A [32]. In intercalated cells, the stimulation of CaSR with an agonist promoted urinary acidification in mice, through the activation of the proton pump enhancing proton secretion in urine [33].

The analysis of the described CaSR functions in the kidney suggests that in the ascending limb and distal convolute tubule CaSR is sensitive to serum calcium because of its location on basolateral membrane of tubular cells. Here, the CaSR modulates calcium reabsorption according to the serum levels of calcium so that its increase may be compensated by the CaSR-mediated inactivation of both passive and active distal calcium reabsorption. Nevertheless, a high calcium excretion is potentially dangerous for the kidney as it increases the probability of calcium-phosphate precipitation inside the kidney. This probability especially increases in the papilla, where the fluid concentration is unusually high. To prevent possible dangerous consequences caused by high calcium excretion, CaSR might induce the modifications counterbalancing the tubular function. In proximal tubules, the antiphosphaturic effect reduces the phosphate load to the distal tubular segments, where CaSR decreases calcium reabsorption. In the collecting duct, CaSR promotes urinary salt dilution and acidification, both necessary to favor the solubility of calcium-phosphate salts [34]. These effects appear to be an integrated system able to prevent calcium-phosphate precipitation in kidney tubules and complications like nephrocalcinosis and the formation of calcium-phosphate stones. This hypothetical system is supported by experiments in tubular cells or tubules from laboratory animals, but their relevance has not yet confirmed in humans [35]. This system, if confirmed, seems to be specifically dedicated to the control of calcium-phosphate precipitation within kidney tubules, which are the principal bivalent ions in human body. However, it could also prevent interstitial precipitation of calcium-phosphate salt in kidney medulla to form Randall's plaque that is an apatite deposit in papillary interstitium on which calcium-oxalate stones may develop after ulceration of urothelium covering it [36,37].

## *CaSR *gene polymorphisms and kidney stones

The relevance of CaSR in calcium homeostasis and our preliminary findings about stone former genotyping led us to study more in depth the possible role of CaSR gene polymorphisms in calcium kidney stones formation. For this purpose, we selected stone forming patients presenting a normal excretion of citrate, because the pathogenetic effect leading to calcium-phosphate precipitation may be predominant compared to the function performed by the precipitation inhibitors, among which urine citrate is one. On the contrary, urine calcium and phosphate promote salt precipitation and CaSR, influencing their tubular handling, may affect urine saturation for their salts [37].

**Figure 3:**
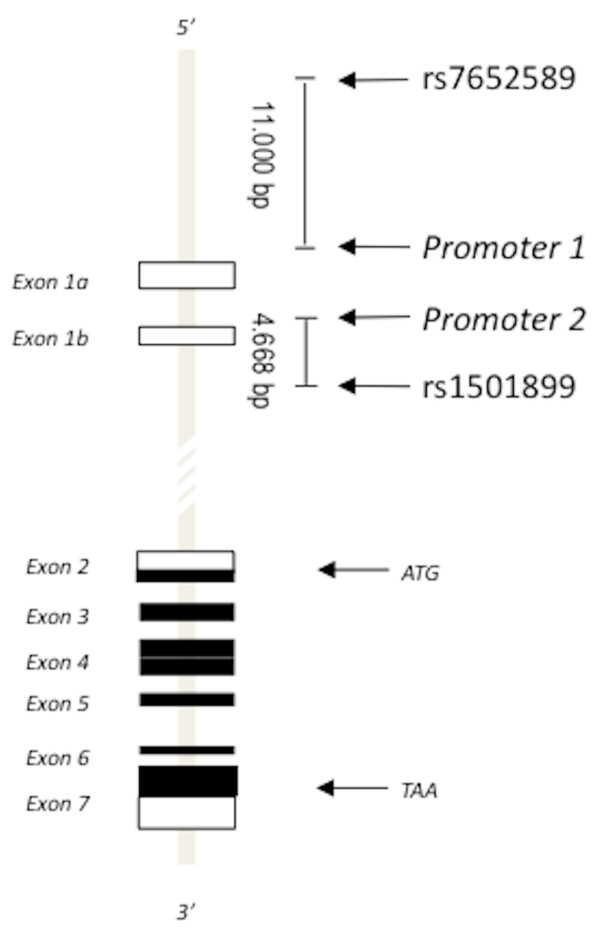
**The *CaSR *gene (chr 3q13.3-21) spans 103 kb and encodes the CaSR protein of 1078 amino acids**. Exons are shown as either unfilled boxes (untranslated regions (UTR)), or filled boxes (protein coding regions). Exons 1a and 1b encode alternative 5'-UTRs. Exons 2-7 encode the CaSR protein. Exon 2 encodes the translation start codon (ATG) and the NH_2_-terminal part of the CaSR. Exon 7 encodes the stop codon (TAA) and the COOH-terminal part of the CaSR. rs7652589 (G > A, position 123371778) is located ~11000 bp upstream of P1 in the 5'-flanking region of the *CASR *gene. rs1501899 (G > A, position 123390018) is located 4668 bp downstream of P2 within intron 1 of the *CASR *gene.

Our study included 463 patients affected by both calcium phosphate and oxalate stone disease of which 312 were normocitraturic and 151 hypocitraturic, and 213 healthy subjects. All of them were genotyped for 21 biallelic polymorphisms mapping the whole *CaSR *gene at a distance of 5-10 kb [9]. The frequency of the minor allele was > 10%. Our results showed that, in addition to the intronic polymorphism rs1501899 (G > A), the polymorphism rs7652589 (G > A) was associated to the normocitraturic stones and to a greater stone rate (figure 3). Their minor alleles were more common in normocitraturic stone formers than in healthy subjects or hypocitraturic stone formers [9].

In order to confirm this study, we replicated it on a second sample of patients presenting primary hyperparathyroidism (PHPT). These patients frequently suffer from stone disease, as they are hypercalciuric (and hypercalcemic) and normocitraturic. This study compared 157 PHPT patients forming kidney stones and 175 PHPT patients not forming stones. Also in this population polymorphisms rs7652589 and rs1501899 were associated with the presence of stones [38].

Polymorphisms rs7652589 and rs1501899 are respectively at 11000 kb before P1 and at 4600 kb after P2 (figure 3). This location led us to assume their possible influence on CaSR genetic translation and CaSR expression. They could directly modify the effect of transcription factors, or be in linkage with other promoter polymorphisms changing CaSR gene promoter function. To confirm this suggestion, we performed a bioinformatic analysis exploring the potential influence of rs7652589 and rs1501899 on specific transcription factors. Findings of our analysis suggested that the minor alleles at these polymorphisms were able to modify the binding sites of specific translational factors and, consequently, the transcription efficacy [9].

Further genotype analysis in normocitraturic patients showed that calcium stones were associated with polymorphisms located within P1, whereas P2 polymorphisms were not associated [39]. These findings suggest that a deficiency in CaSR function may occur in stone formers, caused by a qualitative alteration of the expression and by a defect in P1 activity. Variant alleles in the P1 promoter or transcription regulatory region could support this defect.

## Critical points in the relationship between CaSR and kidney stones

The hypothesis we are proposing is based on association findings and has to be supported by functional data that, confirming it, could clarify the influence of the polymorphisms on the gain or loss of the CaSR expression.

Some data indicate that minor alleles at the associated polymorphism lead to a loss of CaSR expression in the kidney. Supporting this finding, serum levels of PTH were lower in normocitraturic stone formers than in homozygous patients for the variant allele at rs7652589 and rs1501899 [9]. This result agreed with a decreased CaSR expression, as only this condition may lead to a deficient inhibition of the PTH secretion and production in parathyroid glands. Furthermore, we measured CaSR mRNA in the healthy medulla samples from nephrectomies affected by kidney cancer. In homozygous subjects for the minor allele at rs7652589 and rs1501899 we found a lower quantity of CaSR mRNA than in subjects with any other genotype at these polymorphisms [39]. These findings are indirect indications that analyzed polymorphisms give rise to a possible reduction in CaSR expression. Nevertheless, they do not demonstrate their influence on the promoter activity. At the moment, we are carrying out experiments directly estimating the function of the CaSR promoter in the presence of these polymorphisms. Promoter function will be estimated in transfected cells with plasmids containing CaSR gene promoter and luciferase gene. The level of luciferase activity will give an evaluation of the promoter function.

### Missense CaSR gene polymorphisms and stones

Three missense polymorphisms of the CaSR gene have a significant frequency in general population. They are located on exon 7 and are A986S (rs1801725, G > T), R990G (rs1042636, A > G) and Q1011E (rs1801726, C > G). Few studies considering CaSR as a candidate-gene tested the frequency of their alleles in nephrolithiasis. A study was performed in 223 Canadian idiopatic calcium stone formers genotyped for A986S and R990G. It observed significant Hardy-Weinberg disequilibrium at the R990G locus in cases, but not in controls, and attributed this disequilibrium to the association of the polymorphism with stones. Carriers of the 990G allele had an eightfold increase of stone risk [40]. Another study was carried out in a group of 99 Iranian recurrent stone formers and observed a significantly higher frequency of the 986S, 990G and Q1011 alleles in stone formers [41]. Two other studies found a significant association between the 990G allele and stones in Italian patients with primary hyperparathyroidism [42,43]. R990G was also associated with primary hypercalciuria, a disorder predisposing to calcium kidney stones, and the association was observed both in stone formers and in stone free subjects [44,45]. This allele was recognized to cause a gain of CaSR function in transfected embrionic kidney cells HEK-293 [45].

These findings confirm that *CaSR *gene polymorphisms may be involved in calcium nephrolithiasis, but propose an intriguing scenario in which stone formation appears to be favored by an activating polymorphism (R990G) and by polymorphisms decreasing expression of the CaSR gene. Despite their apparently opposite effect, both polymorphisms could predispose to calcium nephrolithiasis. Today, we have no sufficient knowledge to explain this puzzle, that, to be resolved, needs experiments evaluating the CaSR activity in kidney tubules and the functional effect of the variant alleles at these polymorphisms. An initial contribution was given by a study in knockout mice for the calcium channel TRPV5 [33]. TRPV5-/- mice were hypercalciuric, but did not form calcium stones or precipitate; they developed calcium-phosphate precipitate in collecting ducts only after inhibition of H-pump activity that hampers urine acidification. The use of allosteric agonist of CaSR in these mice showed that CaSR stimulated H-pump activity in collecting ducts and allowed to maintain urine stability. TRPV5-/- mice were also polyuric because the stimulation of CaSR by luminal calcium antagonized vasopressin activity in collecting ducts [33].

## Conclusions

Our studies suggest that CaSR is a candidate gene to explain individual predisposition to calcium kidney stones [8]. Stone formation may be favored by a reduced CaSR expression in kidney medulla altering the normal balance among calcium, phosphate, protons and water excretion. This defect may cause the intratubular precipitation of calcium-phosphate crystals and the consequent calcium-phosphate stone formation [46]. It could also predispose to calcium-phosphate precipitation in the papillary interstitium, and the possible consequent formation of the Randall's plaque on which calcium-oxalate stones develop [47].

More extensively, our findings indicate that CaSR could prevent deposition of calcium-phosphate salts in tissues. Immunohistochemistry studies and RT-PCR showed that CaSR expression is decreased in calcified arteries from both uremic and non-uremic patients [48,49]. Studies on rats showed that CaSR agonists (e.g. cinacalcet) could inhibit arterial calcification stimulated by vitamin D [50]. A slower progression of arterial calcifications was observed in uremic hemodialyzed patients on cinacalcet treatment [51] that could explain improving in patient mortality [52].

These data suggest a physiopathological role of CaSR in protecting human tissues against the risk of calcification, a risk induced by the condition of supersaturation in body fluids. The functional importance of CaSR is highlighted by the fact that a CaSR precursor is detectable in unicellular organisms where it senses environmental salinity. The evolution of this molecule provided human beings with a cellular calcium sensor used by kidney and parathyroid cells to keep serum calcium within the normal range. CaSR anticalcification function becomes more complex in human kidney where it contributes to the paracrine regulation of ions and water excretion, thus protecting against calcium-phosphate precipitation. Its anticalcification activity has been hypothesized also in arteries but its mechanism has still to be ascertained and understood.

## Competing interests

The authors declare that they have no competing interests.

## Authors' contributions

All authors have read and approved the final manuscript.

GV, AT and LS drafted the manuscript. LS and AT designed the experiment, drafted the experiments and troubleshooting. AT, ED and AA were responsible for the laboratory assay and performing the experiments. GV, MC, FR, DC and TA participated in clinical study.

## Author's Information

GV MD, nephrologist, Principal Investigator of Clinical Staff

AT PhD in Molecular Medicine

FR MD, Fellow of Nephrology

TA MD, nephrologist

MC MD PhD, nephrologist

AA biologist, PhD student in Molecular Medicine

ED PhD, Nutritionist

DC MD, nephrologist

LS PhD, Principal Investigator of Experimental Staff
